# Endotoxin-Free Outer Membrane Vesicles for Safe and Modular Anticancer Immunotherapy

**DOI:** 10.1021/acssynbio.4c00483

**Published:** 2025-01-07

**Authors:** Mei-Yi Chen, Ting-Wei Cheng, Yi-Chung Pan, Chung-Yuan Mou, Yun-Wei Chiang, Wan-Chen Lin, Che-Ming Jack Hu, Kurt Yun Mou

**Affiliations:** † Chemical Biology and Molecular Biophysics Program, Taiwan International Graduate Program, Academia Sinica, No. 128, Sec. 2, Academia Rd., Nangang (Nankang) Dist., Taipei City 115201, Taiwan; ‡ Institute of Biomedical Sciences, Academia Sinica, Taipei 11529, Taiwan; § Department of Chemistry, National Tsing Hua University, Hsinchu 300044, Taiwan; ∥ Department of Chemistry, National Taiwan University, Taipei 10617, Taiwan

**Keywords:** outer membrane vesicle (OMV), nanoparticles, bacteria engineering, lipopolysaccharides (LPS), immunotherapy

## Abstract

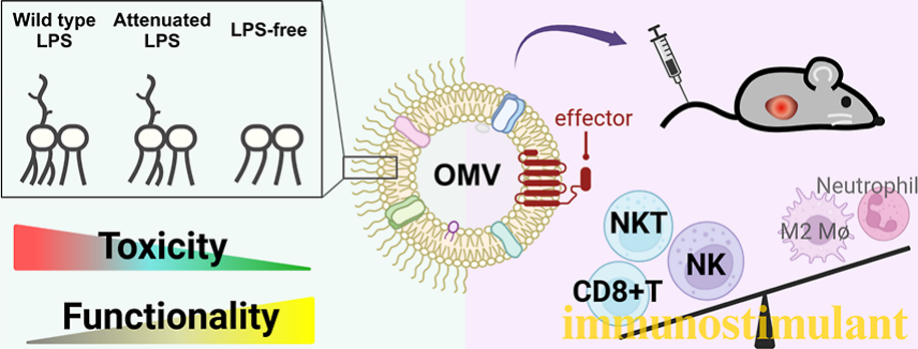

Bacterial outer membrane vesicles (OMVs) have emerged as promising vehicles for anticancer drug delivery due to their inherent tumor tropism, immune-stimulatory properties, and potential for functionalization with therapeutic proteins. Despite their advantages, the high lipopolysaccharide (LPS) endotoxin content in the OMVs raises significant safety and regulatory challenges. In this work, we produce LPS-attenuated and LPS-free OMVs and systematically assess the effects of LPS modification on OMVs’ physicochemical characteristics, membrane protein content, immune-stimulatory capacity, tolerability, and anticancer efficacy. Our findings reveal that LPS removal increased the maximal tolerated dose of the OMVs by over 25-fold. When adjusted for comparable safety profiles, LPS-free OMVs exhibit superior anticancer effects compared with wild-type OMVs. Mechanistic investigations indicate that the LPS removal obviates immune cell death caused by LPS and reduces the negatory effects of wild type of OMVs on tumor immune cell infiltrates. We further show the functionality of the LPS-free OMV through the incorporation of an IL-2 variant protein (Neo-2/15). This functionalization augments OMV’s ability of the OMV to inhibit tumor growth and promote lymphocyte infiltration into the tumor microenvironment. This study presents a safe and functionalizable OMV with improved translational prospect.

## 1 Introduction

Outer membrane vesicles (OMVs) secreted from the outer membrane of Gram-negative bacteria have been broadly adopted as anticancer immunotherapeutic nanocarriers, as the vesicles can stimulate immune responses through the presence of various pathogen-associated molecular patterns such as lipopolysaccharides (LPS, commonly referred to as endotoxins), virulence proteins, and nucleic acids.^1−3^ Intravenous administration of OMVs has been shown to induce tumor-targeted delivery, which can in turn lead to tumor eradication through the induction of cytokines associated with tumor-infiltrating lymphocytes.^4^ OMVs’ functionalizability through various genetic manipulation techniques also offers broad modularity for advanced nanocarrier designs, allowing for surface protein display toward vaccine and targeted drug delivery applications.^5−7^ Although the features highlight the potential of OMVs for cancer treatment and other therapeutics and vaccine development, the highly reactogenic nature of LPS remains a major safety and regulatory concern that impedes the translational development of the carrier.

The LPS content on the OMVs’ can function as a double-edged sword toward anticancer nanomedicine development. Although LPS in the tumor microenvironment can stimulate innate immunity by activating toll-like receptor 4 (TLR4) and trigger cytokine-mediated tumor suppression,^8^ exposure in the bloodstream can lead to excessive levels of systemic cytokines, which can cause severe fever and septic shock.^9^ LPS is also linked to molecular pathways that can trigger pyroptosis,^10^ apoptosis, and necroapoptosis,^11^ which can lead to immune cell death and immune tolerance. In addition, LPS has also been shown to promote cancer proliferation and metastasis in various cancer types by enhancing glycolysis through the NF-kB/Snail/HK3 signaling axis.^12^ These negatory attributes of LPS prompted us to develop endotoxin-free OMVs with the aim of enhancing the biological vesicles’ translational prospect.

In the present work, we prepared LPS-attenuated and LPS-free OMVs using genetically modified bacterial vectors and examined how LPS modification modulates the physicochemical properties, immune stimulatory activities, tolerability, and anticancer efficacy. We show that the removal of LPS enhances OMV’s intravenous tolerability of the OMV, thus enabling high-dose treatment for robust tumor suppression. Mechanistic interrogation reveals that LPS-free OMVs remain immune stimulatory and facilitate tumor immune cell infiltration conducive to tumor suppression. In addition, we observe evidence that removal of LPS can ameliorate LPS-mediated immune cell death. Lastly, we show functionalized LPS-OMVs can be produced through genetic engineering of LPS-modified *Escherichia coli*. As a proof of principle, we show the functionalization of an OMV with a Neo-2/15 cytokine, which enhances the immune stimulatory function and anticancer efficacy of LPS-free OMVs.

## 2 Results and Discussion

### 2.1 Production and Characterization of LPS-Attenuated and LPS-Free OMVs from Genetically Modified *E. coli*

**Figure 1:**
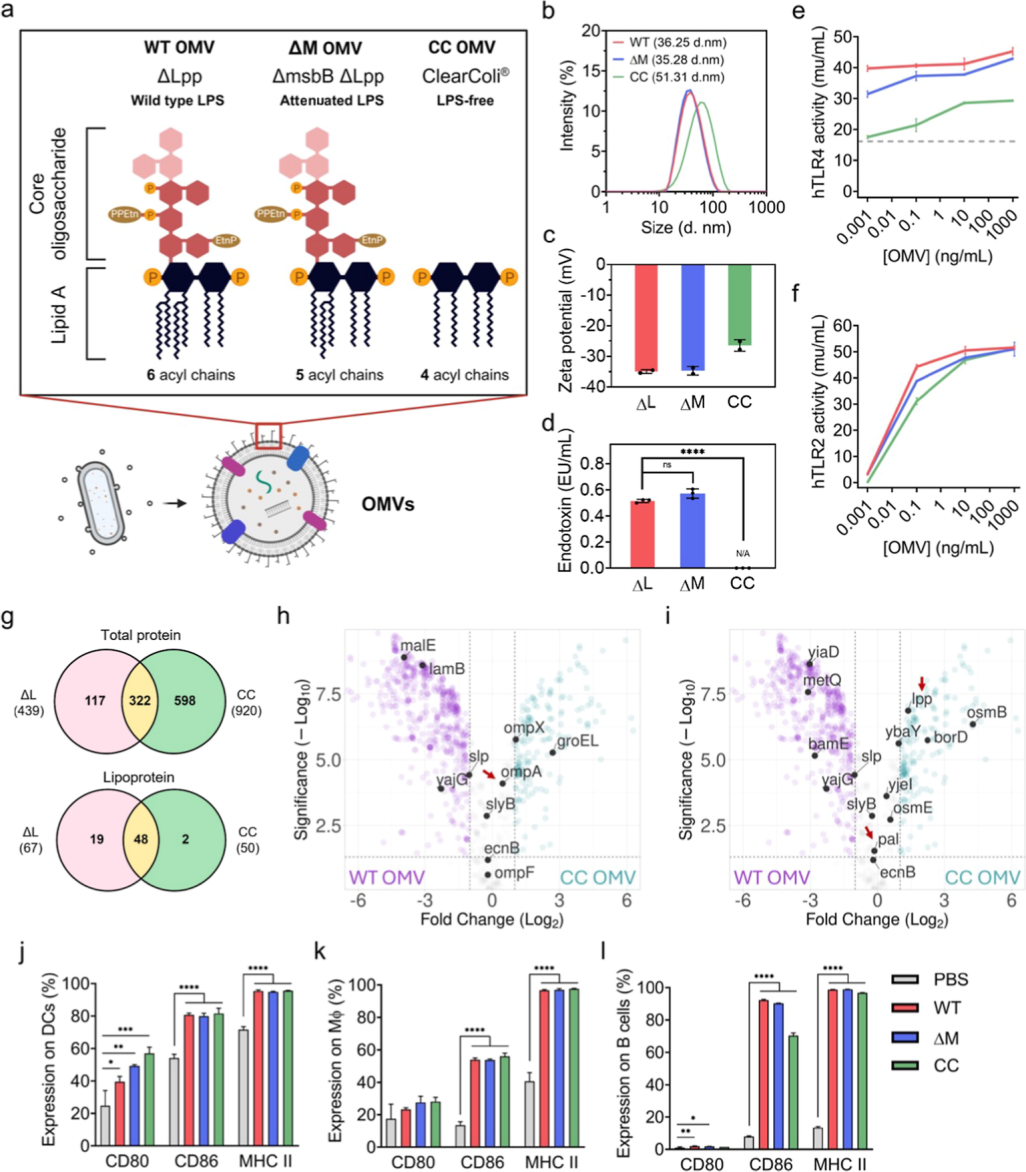
Characterization of lipopolysaccharide (LPS)-attenuated and LPS-free OMVs from genetically engineering *E. coli*. (a) Schematic diagram illustrating the distinctive LPS structures on OMV variants. (b) Size distribution and (c) zeta potential of OMVs measured by dynamic light scattering (DLS). (d) Endotoxin levels were quantified using the limulus amebocyte lysate (LAL) test. OMV variants at 1 pg/mL were used for the assay. Readings for 1 pg/mL CC OMVs were equivalent to the blank well and thus considered as not detectable (N/A). (e) hTLR4 and (f) hTLR2 activities represented as the activation level of NF-kB signaling pathway in HEK-blue-hTLR2 and HEK-blue-hTLR4 reporter cell lines. Dash lines represent background signals. (g) Venn diagram illustrating proteins identified in ΔL (red), and CC OMV (green). (h) Differential protein abundance and (i) lipoprotein (Lpp) abundance comparison between ΔL and CC OMVs. Top 10 proteins/Lpps with the highest abundance identified in ΔL OMVs are indicated by black dots. Major TLR2 agonists, such as Pal and Lpp, are highlighted with red dots, and OmpA is marked with a red arrow. Minor TLR2 agonists are indicated with blue arrows. (j–l) OMVs induced the maturation of splenic antigen-presenting cells regardless of LPS structures. The splenocytes were harvested from C57BL/6 mice and coculture with medium or indicated OMVs (5 μg) for 48 h before flow cytometry analysis. Antigen-presenting cells, including (j) dendritic cells (DCs), (k) macrophage (MΦ), and (l) B cells were analyzed by flow cytometry. CD80, CD86, and MHC II surface molecules were stained as maturation markers. Data were presented as the mean ± SD. Statistical significance was analyzed by a one-way ANOVA with a Tukey’s multiple comparisons test. ns, not significant, *P < 0.05, **P < 0.01, ***P < 0.001, and ****P < 0.0001.

The LPS structure of *E. coli* BL21(DE3) includes core oligosaccharides and lipid A. A typical *E. coli* lipid A moiety comprises hexa-acyl chains and a phosphorylated disaccharide backbone.^13^ We generated three OMV variants containing different LPS structures (Figure 1a). The first OMV variant, possessing intact hexa-acyl lipid A with full endotoxin activity, was isolated from the ΔLpp strain (denoted as ΔL OMVs). The Lpp gene encodes Braun’s lipoprotein (Lpp or murein Lpp), which links the outer membrane to the peptidoglycan layer and maintains membrane integrity. The removal of the Lpp gene improves the yield of OMVs.^14^ The second variant (denoted as ΔM OMVs), generated from the ΔmsbBΔLpp strain, exhibits reduced TLR4 activation capacity. The deletion of the msbB gene, which encodes for lipid A biosynthesis myristoyltransferase, is a commonly adopted LPS-attenuation strategy that generates penta-acyl lipid A-tether LPSs.^15^ The third OMV variant was isolated from the endotoxin-free (or LPS-free) strain ClearColi BL21(DE3) (abbreviated as CC OMVs), which is deprived of seven genes associated with LPS synthesis.^16^ This strain produced OMVs containing tetraacylated lipid A (lipid IVa) with no core oligosaccharide region. We conducted comparative studies between WT OMVs and ΔL OMVs and demonstrated that their properties are largely similar, particularly at higher doses, where distinct differences become less pronounced (Figure S1). Additionally, the sterility of the OMV preparations was confirmed to ensure that subsequent experiments would not be compromised by contamination.

To characterize the physical properties of the OMV variants, we determined the particle size and zeta potential of the OMVs using DLS analysis. The ΔL OMV and the ΔM OMVs were similar in average diameter at 36.25 and 35.28 nm, respectively. On the other hand, CC OMVs had a larger average diameter at 51.31 nm, presumably due to reduction in steric stabilization as a result of LPS removal (Figure 1b). LPS reduction also bestowed CC OMV a more positive zeta potential at −26.5 mV as compared to ΔL and ΔM OMVs (∼−35 mV) (Figure 1c). As prior studies have reported that size differential between 30 and 50 nm shows negligible influence on tumor accumulation,^17,18^ the minor size variation between the different OMVs is overlooked in subsequent analyses.

The endotoxin levels of the different OMVs were measured and compared to the purified LPS standard extracted from the pathogenic *E. coli* strain O111: B4 using both a BCA assay and a LAL test. At an identical protein concentration (1 pg/mL OMV), ΔL OMVs and ΔM OMVs exhibited endotoxin levels of 0.515 ± 0.013 EU/mL and 0.572 ± 0.036 EU/mL, respectively, while CC OMVs showed no detectable signal (Figures 1d and S2). These results confirm the successful use of ClearColi BL21(DE3) for generating OMVs devoid of LPS content.

To examine the immune stimulatory activity of the OMV variants against TLR4 or TLR2, which are putative TLRs for the recognition of bacterial cell wall components,^19^ we measured the OMV-stimulated hTLR2 or hTLR4 activity by the secreted alkaline phosphatase (SEAP) reporter system. HEK-Blue-hTLR2 and HEK-Blue-hTLR4 transfected cell lines are engineered to express the SEAP gene under the control of an NF-κB-responsive promoter. Activation of TLR2 or TLR4 in these cells triggers the secretion of SEAP in the culture medium. The activity of SEAP is subsequently quantified using a luminescent substrate. The results demonstrated that ΔL OMVs elicited the highest level of TLR4 signaling, with a decreased activation observed for ΔM OMVs. Notably, although Lipid IVa is reported as an antagonist for human TLR4,^20^ we detected hTLR4 activity in HEK-blue-hTLR4 cells when cocultured at higher concentrations of CC OMVs, albeit at relatively low levels (Figure 1e). This suggests that other components may contribute to the signal, given that NF-κB can be activated by various upstream factors.^21^ Additionally, the background signal in hTLR4 experiments remained high, even at doses as low as 1 pg. This background signal was defined using the control cell line HEK-Blue-Null2, which expresses the SEAP reporter gene under the control of the IL-12 p40 minimal promoter. It is well-established that dysregulated TLR4 signaling is associated with sepsis caused by Gram-negative bacterial infections.^22^ Although the endotoxin units of CC OMVs can be measured by the LAL test, the SEAP assay indicated that they do not efficiently stimulate the TLR4 signaling pathway (Figure 1d,e). These results support the reduced safety concern of CC OMVs in eliciting cytokine storm and sepsis and corroborate CC OMVs’ improved translational prospect.

In contrast to the variations in hTLR4 stimulation, the OMV variants showed similar stimulatory activity against hTLR2 (Figure 1f). As hTLR2 is activated primarily by Lpps on bacterial cell walls,^19^ the robust hTLR2 stimulatory activity of CC OMV prompted us to examine the protein composition in the OMV variants through label-free quantitative proteomics analysis. The SDS-PAGE profiles of ΔL OMVs and ΔM OMVs were similar; thereby, we compared ΔL and CC OMVs (Figure S3). The analysis identified 439 proteins in ΔL OMVs and 920 proteins in CC OMVs. Among these, 50 Lpps were identified in CC OMVs and 67 Lpps in ΔL OMVs (Figure 1g). A volcano plot analysis between ΔL OMVs and CC OMVs revealed that 5 of the top 10 identified proteins (OmpA, OmpF, OmpX, slyB, and ecnB) were expressed at comparable abundance between the two OMV variants (Figure 1h and Tables S1 and S2). For the top 10 identified Lpps in OMVs, ΔL and CC OMVs also showed similar expression of 6 Lpps, including pal, osmE, slyB, yjel, slp, and ybaY (Figures 1i, S4 and Tables S3 and S4). Of the prominent TLR2 agonists, such as pal and OmpA,^23−27^ CC OMVs express comparable protein abundance as ΔL OMVs. These proteomic findings support the observation that ΔL and CC OMVs induce similar levels of hTLR2 signaling. Furthermore, the larger protein repertoire harbored by CC OMVs may allow them to introduce more exogenous proteins and antigenicity to tumor cells,^28^ potentially benefiting therapeutic outcomes through broadened T cell cross-reactivity.^29−31^

Lastly, given that OMVs’ potent stimulatory activity for maturing antigen presenting cells (APCs) is frequently leveraged for OMV-based vaccine designs and may contribute to their anticancer effect,^5,7,32^ we examined the OMV variants upon incubation with APCs derived from mouse splenocytes. Expression of activation markers 48 h following incubation of mouse splenocytes with the different OMVs showed that all of the OMV variants elevated activation markers, such as CD80, CD86, and MHCII on DCs and macrophages (MΦ) (Figure 1j,k). Curiously, LPS attenuation and removal enhanced CD80 expression on DCs, presumably due to reduction of LPS-mediated immunosuppressive effect.^33−36^ Examination of B cells showed that the different variants upregulated CD86 and MHCII (Figure 1l), highlighting robust immune stimulatory activity by the LPS-modified OMVs.

### 2.2 Antitumor Activity of Wild-Type, LPS-Attenuated, and LPS-Free OMVs

**Figure 2:**
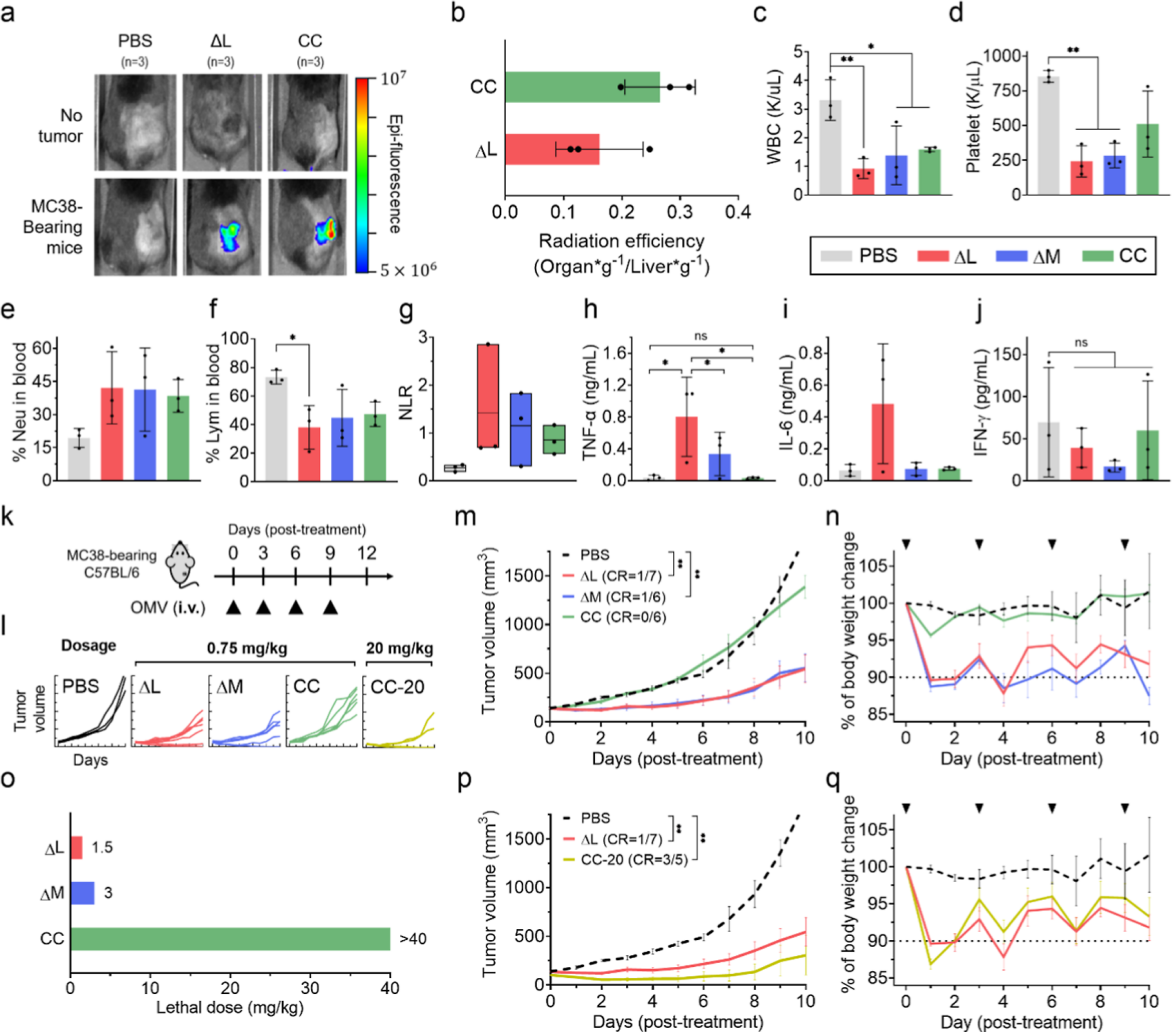
Evaluation of OMV variants for intravenous anticancer treatment. (a) Mice were subcutaneously inoculated with MC38 murine colon adenocarcinoma cells at the right flank. When the tumor volume reached 150 mm^3^, CellMask-labeled OMV variants were intravenously injected into WT and MC38-bearing mice to assess tumor targeting. Representative images were captured 5 h postadministration. (b) Quantification of OMV tumor accumulation by fluorescence measurement. Results were obtained from three independent experiments (*n* = 3) (c–j). The indicated OMVs (15 μg) or PBS were intravenous injected into C57BL/6 mice when the tumor volumes reached 250 mm^3^. After 48 h, blood was collected for complete blood count (CBC) analysis (c–g), and tumors were harvested to examine intratumoral cytokine levels (h–j). The isolated tumors were homogenized and cytokine concentrations were measured using the BD cytometric bead array (CBA) mouse inflammation kit (*n* = 3). Statistical significance was calculated via one-way ANOVA with a Tukey’s multiple comparisons test. **p < 0.01, *p < 0.05. (k) Schematic diagram of the animal tumor model and OMV treatment regimen. C57BL/6 mice were subcutaneously inoculated with murine colon cancer MC38 cells. Treatment with PBS or indicated OMVs (0.75 mg/kg or 20 mg/kg) was delivered via intravenous injection twice, with 3 day intervals, when tumor volumes reached about 100 mm^3^. 20 g adult mouse will receive an OMV dose of 15 μg. (l,m) Tumor volume and (n) mouse body weights were monitored over the course of OMV treatment. (o) Determination of the maximal tolerated dose (MTD) for OMV variants (*n* = 5). (p) Comparison of tumor volumes between CC OMV (20 mg/kg) and ΔL OMV (0.75 mg/kg) treatments at their respective 50% MTD. (q) Mice weight change upon treatment with CC OMV (20 mg/kg) and ΔL OMV (0.75 mg/kg) treatments at their respective 50% MTD. Data are presented as the mean ± SEM. Statistical significance was calculated via two-way ANOVA with a Tukey’s multiple comparisons test. **p < 0.01, *p < 0.05.

Given the previously demonstrated tumor-targeting ability of OMVs upon intravenous administration,^4^ we investigated whether the removal of LPS might affect this capability (Figure 2a). Fluorescence quantification via IVIS imaging following intravenous injection of fluorophore-labeled OMVs revealed that CC OMVs did not compromise tumor targeting compared to ΔL OMVs (Figure 2b). Marginal improvement in tumor accumulation was observed for CC OMVs, which may be attributed to decreased phagocytic clearance as a result of lower inherent immunogenicity of CC OMVs.

To evaluate the systemic inflammatory response induced by the OMV variants, we administered a 0.75 mg/kg dose intravenously and collected blood and tumor samples 48 h post-treatment. Inflammation was assessed using CBC tests and BD CBA assays. CBC tests revealed that all OMV treatments induced leukopenia (Figure 2c), with ΔL and ΔM OMVs specifically causing neutropenia (Table S5) and thrombocytopenia (Figure 2d). OMV increased the neutrophil percentage in the blood (Figure 2e), while the ΔL of the OMVs significantly reduced lymphocyte percentages (Figure 2f). Of note, the neutrophil-to-lymphocyte ratio (NLR), an indicator associated with early sepsis and poor prognosis in immunotherapy patients,^37−39^ was highest with ΔL OMV treatment, which can be a result of severe systemic inflammation due to the endotoxin components (Figure 2g). Correspondingly, TNF-α and IL-6 levels in the tumor were highest in the ΔL OMV treatment group (Figure 2h,i). Although the IFN-γ levels showed no statistically significant differences among the groups (Figure 2j), the ΔM OMV treatment was observed to decrease IFN-γ levels in the tumor. This suggests that LPS-rich OMVs may have a negative impact on immunity within the tumor microenvironment.

Next, to examine the OMV safety and antitumor effect of the OMVs, we intravenously injected the different OMVs at 0.75 mg/kg 4 times over a 9 day interval into MC38 tumor-bearing mice and recorded the weight loss and tumor growth curves (Figure 2k). Although ΔL and ΔM OMVs resulted in more robust tumor suppression than CC OMV (Figure 2l,m), the CC OMV proved to be significantly more tolerable, as its administration at 0.75 mg/kg did not induce body weight change (Figure 2n). These findings highlight the delicate balance between anticancer activity and intravenous tolerability upon modulating LPS content in OMVs. In an intratumoral treatment setting, LPS-free OMVs also exhibited improved tolerability with reduced tumor suppressive activity at equivalent dosing to ΔL and ΔM OMVs (Figure S5).

In light of the safety enhancement observed with CC OMVs, we then examined the MTD of the different OMV variants. Animal studies indicated that the lethal doses for ΔL and ΔM OMVs were 1.5 and 3 mg/kg, respectively, whereas LPS removal significantly enhanced CC OMV tolerability. For CC OMV, a maximal dosing at 40 mg/kg was examined, as the OMV solution became too viscous for injection above 40 mg/kg. Upon intravenous administration of CC OMV at 40 mg/kg, no mouse death was observed, and the injection did not induce any observable physical discomfort (*n* = 5). To further explore the anticancer potential of CC OMVs, we subsequently increased the dose of CC OMVs from 0.75 to 20 mg/kg, which corresponded to half of its observed MTD. In comparison to ΔL OMVs at its 50% MTD of 0.75 mg/kg, CC OMVs at 20 mg/kg improved tumor suppression with a higher percentage of complete responders (1/7 CR for ΔL OMVs at 0.75 mg/kg and 3/5 CR for CC OMVs at 20 mg/kg) (Figure 2p,q). Examination of weight loss further showed comparable tolerability between ΔL of the OMV at 0.75 mg/kg and CC of the OMV at 20 mg/kg. These results support the improved safety profile of LPS-free OMVs and highlight their wider therapeutic window for anticancer applications.

The observed weight loss was comparable between those of high-dose CC OMVs (CC-20) and LPS-containing OMVs (Figure 2n,q), suggesting that systemic inflammation levels may also be similar. However, the mechanisms underlying these adverse inflammatory responses are likely distinct. Structural studies have demonstrated that lipid IVa, the modified LPS in CC OMVs, disrupts TLR4 dimerization and impairs downstream TLR4 signaling.^15^ This suggests that other components within CC OMVs, including Lpps (acting as TLR2 agonists),^1^ bacterial DNA (acting as TLR9 agonists),^40^ bacterial rRNA (acting as TLR13 agonists),^41^ and immunogenic proteins such as GroEL,^42,43^ are likely responsible for activating the immune response.

### 2.3 Examination of Tumor Immune Cell Composition Following Intratumoral Treatment with Wild-Type, LPS-Attenuated, and LPS-Free OMVs

**Figure 3:**
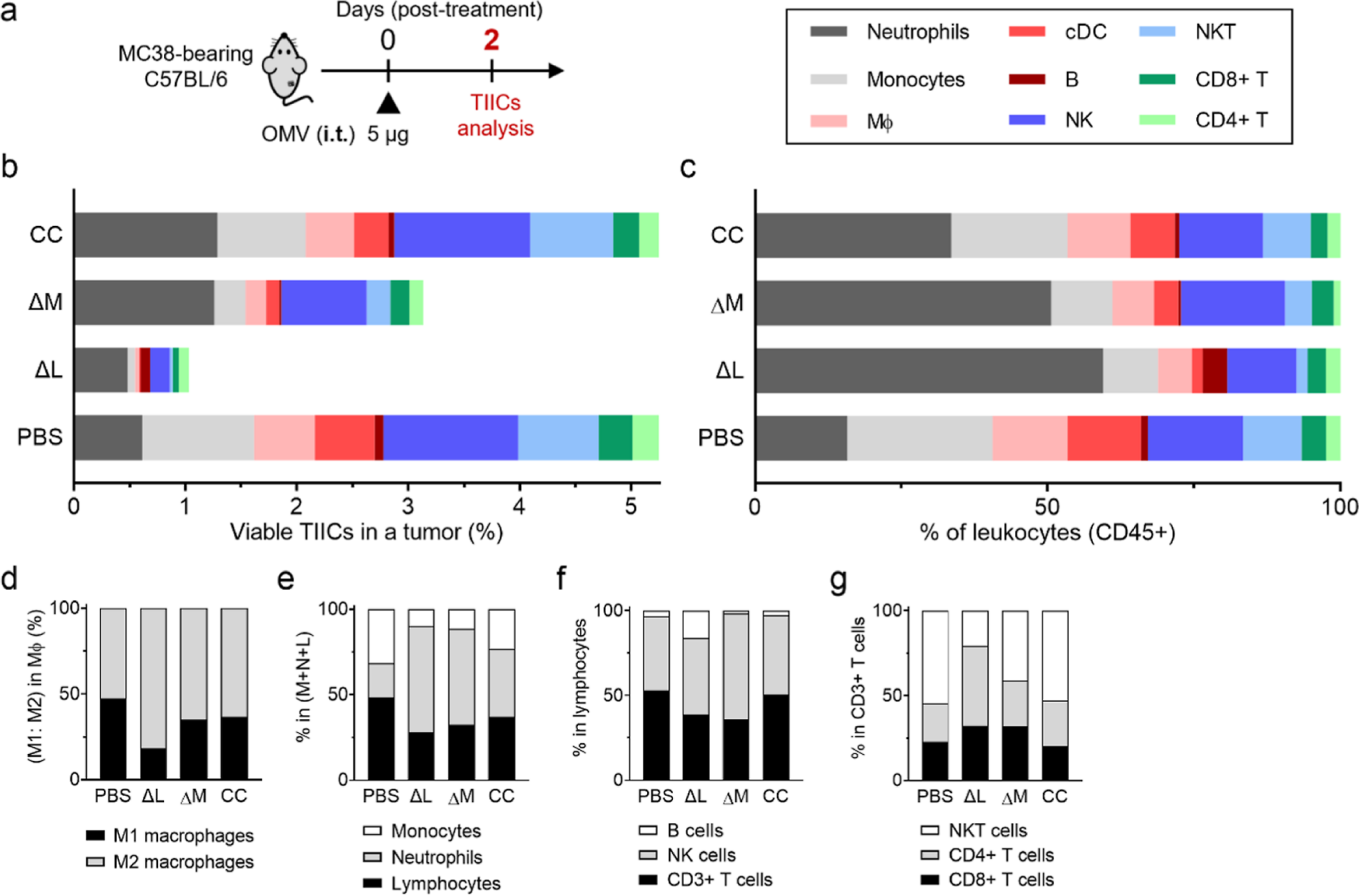
Analysis of TIICs following intratumoral treatment of OMV variants. (a) Schematic diagram illustrating an MC38 tumor-bearing mouse model for intravenous treatment by OMV variants and the time point of TIIC analysis. (b) Absolute immune cell counts in tumors. Total cell counts were normalized to 100,000 cells for every condition, then TIIC were weighted by day-2 tumor volume to reflect actual TIIC number in a tumor. (c) Percentage of TIICs in viable leukocytes (CD45+). TIICs includes neutrophils (Neu), monocytes (Mono), MΦ, conventional DCs (cDCs), B cells, NK cells, NKT cells, CD8+ T cells, and CD4+ T cells. (*n* = 2) (d–g) Percentage of cell subtypes in each group for comparing the change of immune population: the ratio of M1 and M2MΦ (d), the population change of Mono, Neu, and lymphocytes (e), lymphocytes (B cells, NK cells, and CD3+ T cells) (f), and CD3+ T cells (CD4+ T cells, CD8+ T cells, and NKT cells) (g).

The substantial increase in tolerability prompted further investigation into the impact of the variants of the OMV on tumor-infiltrating immune cells (TIICs). On day 2 post a 5 μg intratumoral OMV treatment, TIICs were analyzed using multicolor flow cytometry (Figures 3a and S6). Comprehensive TIIC analysis showed that the immune cells were altered by the different OMV treatments in terms of both cellularity and composition (Figure 3b,c). As both ΔL OMVs and LPS-attenuated ΔM OMVs induced rapid tumor shrinkage and hemorrhagic necrosis as a result of high TNF-α induction, significant reductions in TIICs were observed. Both ΔL and ΔM OMV treatments reduced the number of viable leukocytes within the tumor, highlighting extensive cell death induced by LPS. CC OMV in contrast showed lower influence on TIICs. Upon TIIC analysis following normalization to the total number of CD45+ leukocytes, we observed that all of the OMVs tend to reduce monocytes, MΦ, conventional DCs (cDCs), and CD3+ T while increasing NK cells, B cells, and neutrophils (Figure 3b). Of particular note, neutrophils were present as the major immune cell population among all OMV treatment groups, which is indicative of the overall inflammatory nature of the OMVs. In addition, ΔL OMV treatment increased the number of B cells in the tumors, which may be attributed to LPS’s well-documented mitogenic property on B cells.^44^ These results show that high levels of inflammation can disrupt the delicate balance required for effective immune responses against tumors, and LPS-removal in CC OMV reduced the overall effect on TIICs.

We further examined the polarization of M1 and M2MΦ 2 days following intratumoral OMV administration and observed that ΔL OMV induced M2MΦ polarization, whereas ΔM and CC OMV attenuated this effect (Figure 3c). This finding contrasts with the M1MΦ polarization typically associated with LPS stimulation.^45^ It may instead be attributed to refractory and repair mechanisms activated in response to tissue damage caused by hyperimmune activation.^46^ The treatment of ΔL OMV and ΔM OMV also led to a smaller T lymphocyte population (Figure 3d), which can be attributed to LPS-mediated T lymphocyte suppression.^11,47^ In contrast, the populations of B cell and CD4+ T cell increased following ΔL of the OMV treatment (Figure 3e,f). It has been reported that LPS-stimulated monocytes and B cells can lead to an increase in Tregs and the production of prostaglandin E2 (PGE2), which collectively suppress T cell proliferation and cytokine production.^48,49^ In addition, Parekh et al. demonstrated that B cells activated by LPS induce the anergy of CD8+ T cells.^50^ The apoptosis of cancer cells induced by LPS has been reported to the release of chemotactic factors that attract neutrophils into the tumor microenvironment.^51^ While neutrophils are generally known for their role in the immune response against infections, they can also exhibit pro-tumorigenic activities. These activities include the suppression of tumor-infiltrating T cells, which are crucial for antitumor immunity.^52^ Consequently, the presence of neutrophils within the tumor can facilitate tumor progression and immune evasion by inhibiting the antitumor functions of T cells. At a later time point following intratumor injections of the OMVs, we observed that CC OMVs retained a higher number of TIICs within the tumor microenvironment (Figure S7). Overall, immune cell profiling delineates several negatory attributes of the presence of OMVs on TIICs, which are obviated in LPS-free OMVs.

### 2.4 Splenocytes Proliferation upon OMV Incubation

**Figure 4:**
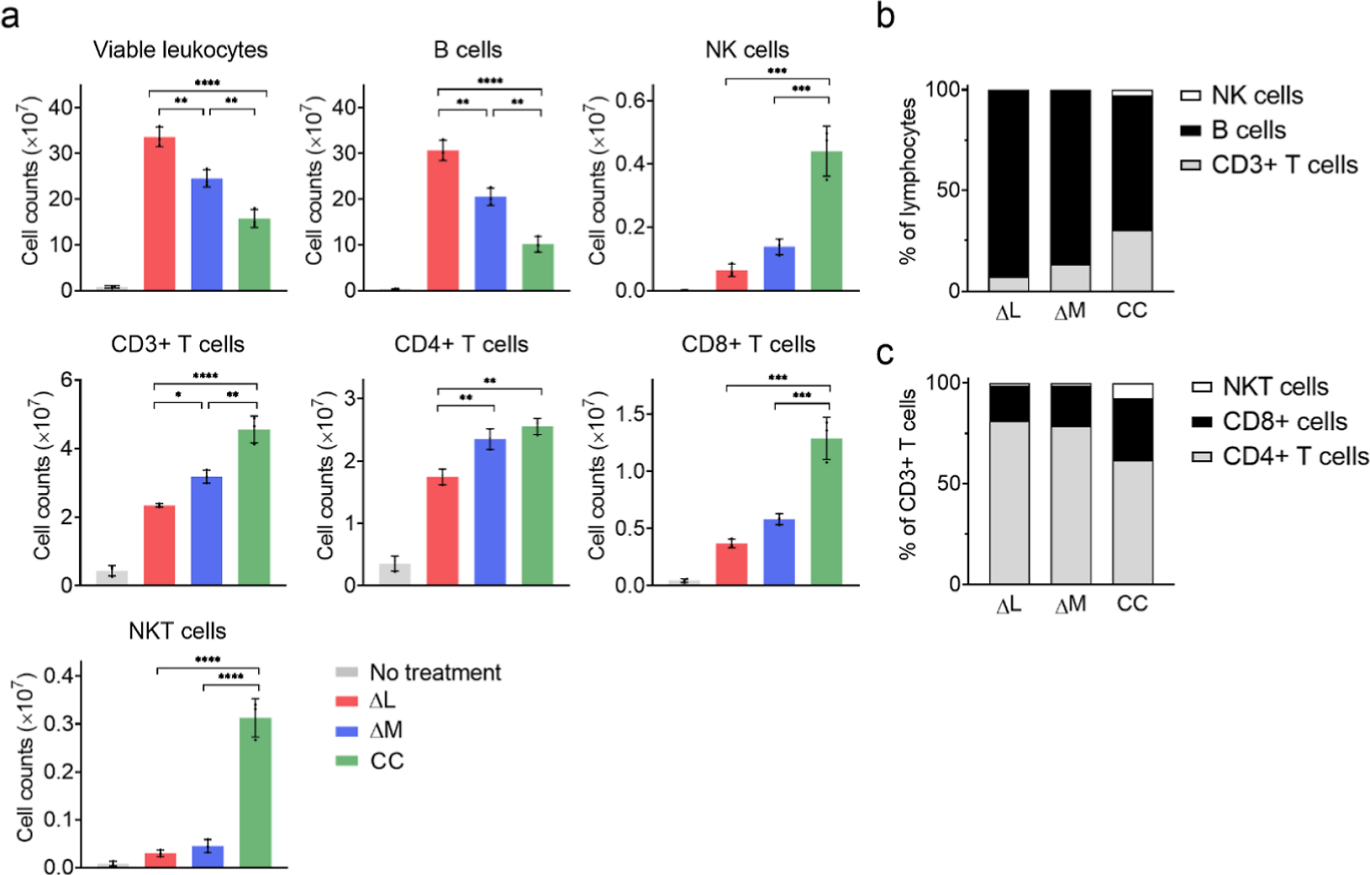
Immune cell stimulation upon splenocyte incubation with OMV variants. (a) Absolute immune cell counts in different OMV treatments. Three mL of splenocyte with a cell density of 2 × 10^6^ cells/mL were incubated with 5 μg OMV variants for 5 days. Cell number were calculated by an automated cell counter, and the immune cell subtypes were analyzed by flow cytometry (*n* = 3). Data are presented as the mean ± SD. Statistical significance was calculated via one-way ANOVA with a Tukey’s multiple comparisons test. *P < 0.05, **P < 0.01, ***P < 0.001, and ****P < 0.0001. (b) Percentage of cell subtype in lymphocyte (B cells, CD3+ T cells, and NK cells). (c) Percentage of cell subtype in CD3+ T cells (CD4+ T cells, CD8+ T cells, and NKT cells).

To further examine how the different OMV variants may exert a differential stimulatory effect on immune cells, we isolated splenocytes from C57BL/6 mice and examined their survival and growth upon OMV incubation. We first examined the overall cytotoxic effect of the OMV variants on splenocytes, and an optimal OMV dose of 1.67 μg/mL that maximizes splenocyte viability was identified for subsequent analysis (Figure S8). Total cell counts were determined using an automated cell counter, and cell compositions were analyzed using flow cytometry. After 5 days of OMV treatment, ΔL of the OMVs were observed to stimulate the highest number of viable leukocytes, with B cells being the predominant immune cell subtype among the viable leukocytes. Conversely, although CC OMVs induced less overall splenocyte proliferation, the absolute counts of lymphocytes, including NK cells, CD4+ T cells, CD8+ T cells, and NKT cells, were the highest (Figure 4a).

Given that LPS’s potent mitogenic property on B cells, its attenuation and reduction in ΔM and CC OMV led to a decrease in the B cell population among the total lymphocyte population (Figure 4b). Accompanying the reduction in the overall B cell population are higher percentages of NK cells, CD8+ T cells, and NKT cells induced by ΔM OMV and CC OMV. It has been previously reported that LPS can trigger lymphocyte exhaustion by stimulating the expression of Tim-3 on CD4+ T cells, CD8+ T cells, and NK cells. This upregulation of Tim-3 has been associated with decreased production of IFN-γ, reduced cytotoxicity of NK cells, and increased apoptosis.^53,54^ These properties help explain the reduced stimulatory property of ΔL OMV and ΔM OMV on lymphocytes (Figure 4c). CC OMV thus shows increased counts of NK cells and CD8+ T cells as a result of the LPS removal.

### 2.5 LPS-Free OMVs Functionalized with IL-2 Variant Shows Enhanced Immune Stimulatory and Anticancer Activity

**Figure 5:**
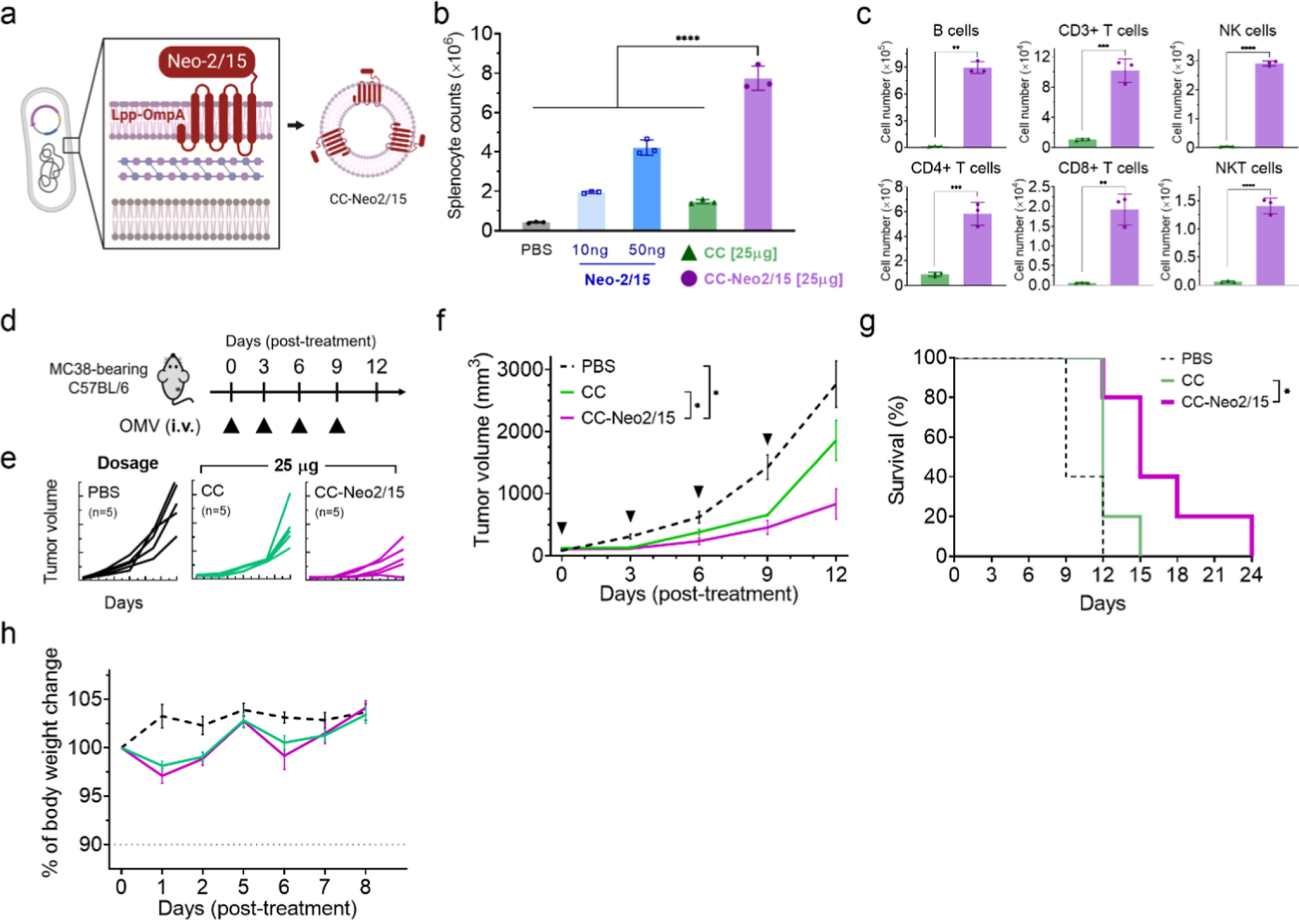
CC OMV functionalization with Neo2/15 for enhanced immune cell stimulation and anticancer efficacy. (a) Schematic of engineered bacteria generating fractionalized OMVs through bacteria surface display systems. (b) 1 mL of mice splenocyte with a cell density of 2 × 10^6^ cells/mL were treated with Neo-2/15or indicated OMV variants. (c) Immune cell population following splenocyte incubation with OMV variants as analyzed by flow cytometry (*n* = 3). Data are presented as the mean ± SD. Statistical significance was calculated via one-way ANOVA with a Tukey’s multiple comparisons test (*P < 0.05, **P < 0.01, ***P < 0.001, and ****P < 0.0001). (d) Schematic illustrating an MC38 tumor-bearing mouse model for intravenous treatment by OMV variants. (e) Individual tumor growth curves and (f) tumor growth curves following OMV treatment. Data were presented as the mean ± SEM. Statistical significance was analyzed by a two-way ANOVA with a Tukey’s multiple comparisons test. (ns, not significant, *P < 0.05, **P < 0.01, and ***P < 0.001.) (g) Kaplan–Meier analysis of mice survival following OMV treatment. Tumor volume larger than 1500 mm^3^ were recognized as death (*n* = 5). Statistical significance was analyzed by a one-way ANOVA (*P < 0.05). (h) Weight change of mice following intravenous OMV delivery.

In light of the improved tolerability, tumor tropism, and inherent immune stimulatory effect of the CC OMV for anticancer immunotherapy, we further demonstrate the modular nature of the OMVs for efficacy enhancement. As a proof of principle, we functionalized CC OMVs with a computationally designed IL-2 variant Neo-2/15 (Figure 5a), which selectively stimulates CD8+ T cells without activating Tregs.^55^ To generate the Neo-2/15-bearing CC OMV (CC-Neo2/15 OMV) and ensure cytokine localization on the surface of the vesicles, Neo2/15 was cloned in conjunction with Lpp-OmpA, encoding a transmembrane domain into the pET3a vector. The transformed *E. coli* containing the pET3a-LppOmpA-Neo2/15-HA plasmid was then cultured for CC OMV isolation. Quantification of the Neo-2/15 on CC-Neo2/15 OMV via Western blotting and ImageJ analysis showed that the functionalized OMVs contained approximately 1.5% of Neo-2/15 (Figure S9). In parallel, we generated purified Neo-2/15 proteins from *E. coli* BL21(DE3) as a control. The stimulatory activity of the cytokine protein was confirmed by a splenocyte proliferation assay (Figure 5b), which showed that coculturing with Neo-2/15 increased the splenocyte count in a dose-dependent manner. At identical OMV dosages, CC-Neo2/15 OMV induced a 5.26-fold increase in splenocyte proliferation assay compared to CC OMV (CC: 1.469 ± 0.113 × 10^6^ cells; CC-Neo215:7.738 ± 0.606 × 10^6^ cells) (Figure 5b), thereby confirming enhancement of immune stimulatory activity of OMV upon cytokine functionalization. Following a 5 day stimulation period, CC-Neo2/15 OMV also showed significant increases in CD3+ T cells [9.6-fold], including CD4+ T cells [6.4-fold], CD8+ T cells [32.5-fold], and NKT cells [22.5-fold], as well as NK cells [67.9-fold] and B cells [73.5-fold] as compared to CC OMV (Figure 5c).

Upon intravenous administration in mouse models bearing MC38 colorectal tumors (Figure 5d), CC-Neo2/15 at equivalent dosing of 25 μg of protein content showed significant enhancement in tumor suppression (Figure 5e,f) (tumor volume at day 12: PBS = 2765 ± 371 mm^3^; CC = 1862 ± 378 mm^3^; and CC-Neo215 = 831 ± 246 mm^3^) and conferred higher survival benefit as compared to CC OMV (Figure 5g). Body weight monitoring showed comparable intravenous tolerability between CC OMV and CC-Neo2/15 OMV as both variants caused less than 3% weight reduction (CC: 98.1 ± 1.01%; CC-Neo215:97.1 ± 1.76%) (Figure 5h). These results highlight the CC OMV as a safe and modular nanocarrier for therapeutic nanoparticle engineering.

## 3 Conclusions

In this study, we systematically investigated the antitumor effects of OMV variants with differing LPS structures (wild-type LPS, attenuated LPS, and LPS-free), focusing on their impact on TIICs and overall therapeutic efficacy.

Our findings revealed that the removal of LPS significantly improved the safety profile of the OMVs, as LPS-free OMVs minimized systemic inflammation and avoided the immunosuppressive effects typically induced by LPS. This improvement in safety markedly increased the tolerability of the OMVs, enabling greater infiltration of T cells and NK cells into the tumor microenvironment. In contrast, LPS-containing OMVs, such as ΔL and ΔM OMVs, elicited severe systemic inflammation and adverse effects, including leukopenia, thrombocytopenia, and an elevated NLR. These toxic effects substantially restricted the therapeutic window of LPS-containing OMVs, highlighting the importance of LPS removal in improving the tolerability of the OMV for clinical applications.

Leveraging the enhanced tolerability of LPS-OMVs and its improved translational prospect, we further showed the platform’s adaptability for cytokine functionalization and cancer immunotherapy. We demonstrated CC OMV functionalization with Neo-2/15, a computationally engineered IL-2 variant designed to selectively stimulate CD8+ T cells over Tregs. CC-Neo2/15 OMVs significantly promoted splenocyte proliferation and increased the populations of key immune cell subsets, including CD4+ T cells, CD8+ T cells, NK cells, and NKT cells. In vivo studies using MC38 tumor-bearing mice revealed that CC-Neo2/15 OMVs achieved superior tumor suppression and extended survival rates compared to nonfunctionalized CC OMVs while maintaining a favorable safety profile with minimal weight loss.

Overall, this study highlights the importance of LPS removal in developing OMVs for clinical applications and demonstrates that functionalizing LPS-free OMVs with cytokines like Neo-2/15 can significantly enhance their antitumor efficacy while maintaining a broader therapeutic index and reducing the risk of systemic inflammation and sepsis, paving the way for safer and more effective immunotherapeutic strategies using OMVs.

## 4 Methods

### 4.1 Cell Line Culture

Mouse MC38 colon cancer cells were cultured in DMEM medium supplemented with Penicillin/Streptomycin solution (PenStrep) (100 U/mL) (Corning, #30-004-CI), 1 mM sodium pyruvate (Gibco, #11360070), nonessential amino acids (NEAA) (Gibco, #11140050), 10 mM HEPES (Gibco, #15630080), and 10% FBS (Corning, #35-010-CV) at 37 °C with humidified 5% CO_2_ supplement. The HEK-blue-hTLR4, HEK-blue-hTLR5, and HEK-blue-Null2 reporter cell lines were purchased from invivogen and were maintained in DMEM supplemented with 10% heat-inactivated FBS (56 °C, 30 min) and PenStrep (100 U/mL) in a humidified incubator at 37 °C and 5% CO_2_.

### 4.2 Animal Source

C57BL/6 mice (6 to 8 weeks old) were obtained from the National Laboratory Animal Center, NARLabs, Taiwan. All animal experiments were conducted under specific pathogen-free conditions and followed the guidelines approved by the Animal Care and Usage Committee of Academia Sinica.

### 4.3 Construction and Expression of Bacterial Display Neo-2/15

Lpp-OmpA and Neo-2/15 were cloned into the pET3a vector using the Gibson assembly cloning procedure [49], incorporating an HA tag at the C-terminal end of Neo-2/15. The linker sequence between Lpp-OmpA and Neo-2/15 is “GGGGSTS”. The DNA sequence for Neo2/15 was obtained from Invitrogen GeneArt gene synthesis services. To generate LPS-free OMVs displaying Neo-2/15 on the surface (CC-Neo215), the plasmid was transformed into the BL21(DE3) ClearColi strain via the heat shock method. The transformed *E. coli* containing the pET3a-LppOmpA-Neo2/15-HA plasmid was cultured in LB Broth (Miller) at 37 °C with shaking at 160 rpm for 24 h without IPTG. The bacterial culture was centrifuged, and OMVs were isolated using the following OMV purification procedure.

### 4.4 OMV Purification

The *E. coli* BL21(DE3) ΔLpp, ΔmsbBΔLpp, and ClearColi strains were cultured overnight in LB broth at 37 °C with shaking at 160 rpm. After overnight incubation, the cell cultures were centrifuged at 6000 rpm at 4 °C for 40 min. The supernatants were then filtered using a 0.45 μm pore size MCE membrane (ChromTech, #MJM4547) to remove any residual bacteria. The filtrate was further processed by passing through a 100 kDa cutoff filter membrane for diafiltration, which allowed the OMVs to be kept in PBS. The concentrated filtrate was subsequently subjected to ultracentrifugation at 45,200 rpm for 3 h at 4 °C to pellet the OMVs. The OMV pellet was resuspended in PBS and stored at −80 °C for long-term storage. The protein concentration was determined by a BCA protein assay kit (ThermoFisher, #23225).

### 4.5 OMV Characterization (Particle Size and Zeta Potential)

The size and zeta potential of the OMVs were measured by DLS analyses using a Malvern Zetasizer Nano-ZS ZEN 3600. The data were analyzed with Zetasizer software 7.11.

### 4.6 Determination of Endotoxin Activity by Limulus Amebocyte Lysate Test

The endotoxin levels of the OMV variants were determined by the Pierce LAL chromogenic endotoxin quantitation kit (ThermoFisher, # A39552).

### 4.7 SEAP Assay

A total of 3 × 10^4^ HEK-Blue-hTLR4 cells or 1.5 × 10^4^ HEK-Blue-hTLR2 cells were seeded in 96-well plates with 200 μL of DMEM per well. HEK-Blue-Null2 cells served as the control cell line. After 24 h of OMV incubation, the supernatant was heat-inactivated at 65 °C for 30 min. SEAP substrates (Cayman, #600183) were then added, and the mixture was incubated at room temperature for 10 min. SEAP activity was quantified using a SpectraMax L Microplate Reader (Molecular Devices).

### 4.8 Proteomics Sample Preparation

Isolated OMVs were lysed using a solution containing 4% SDS, 100 mM Tris–HCl (pH 9), and a 1× protease inhibitor cocktail set III. The cell lysates were heated at 95 °C for 5 min and then sonicated for 15 min using a Bioruptor Plus (Diagenode). After centrifugation at 18,000*g* for 30 min at 4 °C, the supernatant was collected. Approximately 50 μL of the supernatant was mixed with 200 μL of methanol, 50 μL of chloroform, and 150 μL of double-distilled water. After allowing the sample to stay at room temperature for 10 min, the aqueous phase was removed. The sample was then mixed with an additional 150 μL of methanol. The resulting pellet was collected, dried for 20 min, and resuspended in 8 M urea and 50 mM triethylammonium bicarbonate buffer. The samples were reduced with 10 mM DTT, alkylated with 50 mM IAA, and digested by using LysC and trypsin. Following acidification, the supernatant was loaded onto SDB-XC StageTips^56^ and eluted with 80% ACN containing 0.1% TFA. The sample was lyophilized and stored at −20 °C until further LC–MS/MS analysis.

### 4.9 LC–MS/MS Experiments

The sample was loaded onto a trap column (2 cm × 75 μm i.d., symmetry C18) and then separated using a nanoACQUITY UPLC System (Waters, USA) equipped with a 25 cm × 75 μm i.d., BEH130 C18 column (Waters, USA). The separation phase employed a gradient of 5–35% buffer B (buffer A: 0.1% formic acid; buffer B: 0.1% formic acid in acetonitrile) at a flow rate of 300 nL/min, over a total run time of 120 min. Mass spectrometric data were acquired on a high-resolution Q Exactive HF-X mass spectrometer (Thermo Fisher Scientific, Bremen, Germany) operating in a data-dependent mode. Full MS resolution was set to 60,000 at 200 *m*/*z*, with a mass range of 350–1600. dd-MS2 resolution was set to 15,000 at 200 *m*/*z*, with an isolation width of 1.3 *m*/*z* and normalized collision energy at 28%. The LC–MS/MS data were analyzed against the human SwissProt database using the Mascot search engine v.2.6.1 (Matrix Science, UK), with precursor peptide mass tolerance set to 10 ppm and MS/MS fragment tolerance set to 0.02 Da.

### 4.10 Proteomics Data Processing and Statistical Analysis

Raw LC/MS data were converted to an mzML file by MSConvert and processed using FragPipe version 21.1. Database search was performed with the MSFragger search engine through the Uniprot database.^47^ Both protein and peptide levels were filtered by a 1% false discovery rate (FDR). The variable modification settings included oxidation (M) and Acetyl (protein N-term), and the fixed modification setting included carbamidomethyl (C). Peptide identification was enhanced by MSBooster^57^ and validated PSM by Percolator.^58^ Protein identification was performed using ProteinProphet.^59^ Label-free quantification was analyzed by IonQuant,^60^ and the “match between runs” was set as 1 min. The statistical analysis was performed using FragPipe-Analyst version 0.39. The MaxLFQ intensity was normalized using variance-stabilizing normalization, and missing values were imputed by the k-nearest neighbors method. FDR corrected using the Benjamini–Hochberg procedure and a cutoff value by 0.01. The Log2 fold change cutoff value was 1 for the volcano plot.

### 4.11 C57BL/6 Splenocyte Isolation and Primary Splenocyte Culture

Spleens from C57BL/6 mice were harvested and mechanically disrupted in 2 mL of RPMI 1640 medium to isolate the splenocytes. The cell suspension was filtered through a 100 μm cell strainer to achieve a single-cell suspension. Red blood cells were removed using RBC lysis buffer (BioLegend, #420301), followed by centrifugation at 300*g* for 5 min and a PBS wash. The splenocytes were cultured in RPMI 1640 medium supplemented with Pen/Strep (100U/mL), 55 μM 2-mercaptoethanol (Gibco, #21985023), 1 mM sodium pyruvate, NEAA, 10 mM HEPES, insulin-transferrin-selenium (Gibco, #41400045), and 10% heat-inactivated FBS (Corning, #35-010-CV) in a humidified incubator at 37 °C and 5% CO_2_.

### 4.12 Antigen-Presenting Cell Maturation Assay

Splenocytes, at a concentration of 2 × 10^6^ cells/mL, were incubated with 5 μg of OMV variants in a 3 mL volume for 48 h at 37 °C with 5% humidified CO_2_. Following incubation, cells were collected and resuspended in a FACS buffer (PBS with 2% FBS) for flow cytometry analysis. To reduce nonspecific binding to Fc receptors, splenocytes were blocked using purified antimouse CD16/32 antibody prior to staining with markers for immune cell subset identification. Cells were stained at 4 °C for 1 h and washed by PBS three times before being analyzed on an Attune NxT cytometer. Dead cells were detected using the viability dye eFluor 780. Viable DCs [eFluor780(−), CD45(+), CD11c (+)], MΦ [eFluor780(−), CD45(+), F4/80(+)], and B cells [eFluor780(−), CD45(+), CD19(+)] were distinguished based on their surface markers, and APC maturation was assessed through the expression of CD80, CD86, and MHC II. Flow cytometry data were processed by using FlowJoX 10.0.7r2 software. Table S6 lists the antibodies used in this study.

### 4.13 Mouse Antitumor Experiments

Male and female mice (6 to 8 weeks old) were subcutaneously inoculated with 5 × 10^5^ MC38 cancer cells at the right flank. The length and width of tumor were measured with a caliper. The size of the tumor was calculated according to the formula: 0.52[(tumor length + tumor width)/2]^3^. When the tumor size reached about 100–150 mm^3^, the mice received PBS or indicated OMVs via intratumoral or intravenously injection.

### 4.14 Tumor Targeting IVIS Analysis

*E. coli* ΔLpp and ClearColi OMVs were labeled with CellMask Deep Red plasma membrane stain (ThermoFisher, #C10046) through a 1 h incubation at 37 °C. Excess CellMask was removed by washing with PBS at least three times using a 100 kDa MWCO centrifugal filter (3000 rpm for 10 min at 25 °C). Prior to injection, mice were fed a low-fluorescence diet (iVidneo; Oriental Yeast, Tokyo, Japan) for at least 3 days. Mice, both normal and MC38 tumor-bearing, were intravenously injected with 20 μg of CellMask-labeled OMVs. CellMask signals were measured using an IVIS system (Xenogen, #IVIS-200) 5 h postinjection. Ten hours postinjection, mice were euthanized and perfused with PBS to minimize background signals. Liver and tumor tissues were isolated to measure the CellMask signal and the weight of each organ for radiant efficiency calculations. Data were analyzed using Living Image 4.5.2 software.

### 4.15 Cytometric Bead Array Assay

MC38 tumor-bearing mice with tumor sizes exceeding 250 mm^3^ were intravenously injected with either PBS or 15 μg of the OMV variants. After 48 h, the tumors were homogenized in RIPA buffer containing protease inhibitor cocktail (MedChem Express, #HY-K0010P-1) using a hand-held homogenizer. The homogenates were analyzed to determine the levels of intratumoral cytokines. The BD CBA mouse inflammation kit (BD biosciences, #552364) was used for the quantitative measurement of interleukin-6 (IL-6), IL-10, monocyte chemoattractant protein-1 (MCP-1), interferon-γ (IFN-γ), tumor necrosis factor, and IL-12p70 protein levels in the tumors.

### 4.16 TIICs Flow Cytometry Analysis

MC38 tumors were harvested and cut into small pieces in TILs buffer (RPMI 1640 medium consisting of 0.3 mg/mL Collagenase type IV (Sigma, #C5138) and 0.06 mg/mL DNaseI from Bovine Pancreas (Cyrusbioscience)) and were subjected to obtain single-cell suspension by using the gentleMACS Octo Dissociator with Heaters (Miltenyi Biotec), following the manufacturer’s instructions. The homogenates were passed through 100 μm cell strainers to remove large cell aggregates and depleted in the presence of red blood cells by RBC lysis buffer (Biolegend, #420301). The homogeneous tumor-immune mixtures were analyzed on an Attune NxT cytometer. The staining procedures were described in the APC maturation assay. To identify the immune cell subtypes, the following gating strategies were applied: viable leukocytes [eFluor 780(−), CD45(+)], B cells [eFluor 780(−), CD45(+), CD3(−), CD19(+)], DCs [eFluor 780(−), CD45(+), CD11b(+), CD11c(+), MHC II(+)], MΦ [eFluor 780(−), CD45(+), CD11b(+), F4/80(+)], monocytes [eFluor 780(−), CD45(+), CD11b(+), Ly6g(−), F4/80 (−)], neutrophils [eFluor 780(−), CD45(+), CD11b(+), Ly6G(+)], NK cells [eFluor 780(−), CD45(+), CD3(−), NK1.1(+)], NKT cells [eFluor 780(−), CD45(+), CD3(+), CD8(+), NK1.1(−)], and CD4+ T cells [eFluor 780(−), CD45(+), CD3(+), CD4(+), NK1.1(−)]. The total cell counts were normalized to 100,000 cells for every condition (*n* = 2). Flow cytometry data were processed using FlowJoX 10.0.7r2 software. Table S6 lists the antibodies used in this study.

### 4.17 Splenocytes Proliferation Assay

Splenocyte with a cell density of 2 × 10^6^ cells/mL were incubated with the corresponding OMV variants for 5 days. The culture volume and OMV dosages are indicated in Figures 4 and 5, separately. The number of viable splenocytes was assessed by staining with trypan blue, and the cell number was determined using an EVE PLUS Automatic Cell Counter. The immune subtypes were analyzed by an Attune NxT cytometer. To identify the immune cell subtypes, the following surface markers are used: viable leukocytes (eFluor 780(−), CD45(+)), B cells (CD3(−), CD19(+)), NK cells (CD3(−), NK1.1(+)), NKT cells (CD3(+), NK1.1(+)), CD8+ T cells (CD3(+), CD8(+), NK1.1(−)), and CD4+ T cells (CD3(+), CD4(+), NK1.1(−)). The total cell counts were normalized to 100,000 cells for every condition. Table S6 lists the antibodies used in this study.

## Supporting Information Available

Reader values (OD405) of LAL test for endotoxin quantification; comparison of wild-type OMV (derived from BL21(DE3) strain) and ΔLpp OMV (derived from BL21(DE3) ΔLpp strain) to cytokine release, hTLR4 and hTLR2 signaling, and cytotoxicity; SDS-PAGE of OMV variants; volcano plot of ΔL and CC OMV; anticancer effects of LPS-free OMVs; flow cytometric analysis of TIICs; analysis of TIICs, following intratumoral treatment with OMV variants; CCK8 assay for determining the viability of C57BL/6 splenocyte at different OMV variant concentrations; quantification of CC-Neo215 by Western blotting and ImageJ analysis; top 10 proteins identified in ΔL OMVs; top 10 proteins identified in CC OMVs; top 10 Lpps identified in ΔL OMVs; top 10 Lpps identified in CC OMVs; whole blood count (CBC) analysis of 2 day OMV treatment by intravenous administration (15 μg); and antibodies list (PDF)
