# Supplementary material for: Endotoxin-Free Outer Membrane Vesicles for Safe and Modular Anticancer Immunotherapy
Source: ACS Synth Biol. 2025 Jan 7;14(1):148–60. doi: 10.1021/acssynbio.4c00483 (PMC11744915; doi:10.1021/acssynbio.4c00483)
Supplement: Supplementary file 1 — sb4c00483_si_001.pdf [file sb4c00483_si_001.pdf]

Supporting Information

Endotoxin-free Outer Membrane Vesicles for Safe and Modular Anticancer Immunotherapy

Mei-Yi Chen[1,2,3], Ting-Wei Cheng[1,2], Yi-Chung Pan[1,2], Chung-Yuan Mou[4], Yun-Wei Chiang[3], Wan-Chen Lin[1,2,*], Che-Ming Jack Hu[2,*], and Kurt Yun Mou[†]

[1]Chemical Biology and Molecular Biophysics Program, Taiwan International Graduate Program, Academia Sinica, No. 128, Sec. 2, Academia Rd., Nangang (Nankang) Dist., Taipei City 115201, Taiwan

[2]Institute of Biomedical Sciences, Academia Sinica, Taipei, 11529, Taiwan

[3]Department of Chemistry, National Tsing Hua University, Hsinchu, 300044, Taiwan

[4]Department of Chemistry, National Taiwan University, Taipei 10617, Taiwan

[†]The author passed away on August 28th, 2023

Email: wchlin@ibms.sinica.edu.tw and chu@ibms.sinica.edu.tw

Supplementary Figures……………...page S2
Supplementary Tables………………page S11

Supplementary Figures

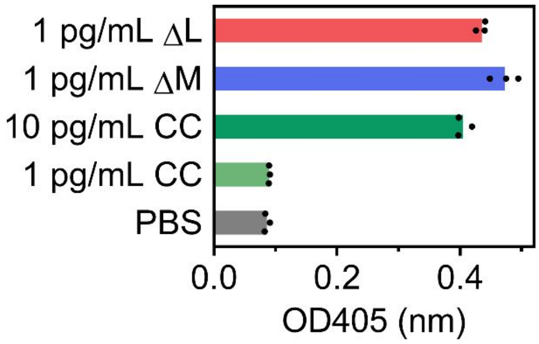

Figure S1: **The reader values (OD405) of LAL test for endotoxin quantification.** OMV variants at 1 pg/mL were used for the assay. The readings for 1 pg/mL CC OMVs were equivalent to the blank well and thus considered as not detectable (N/A).

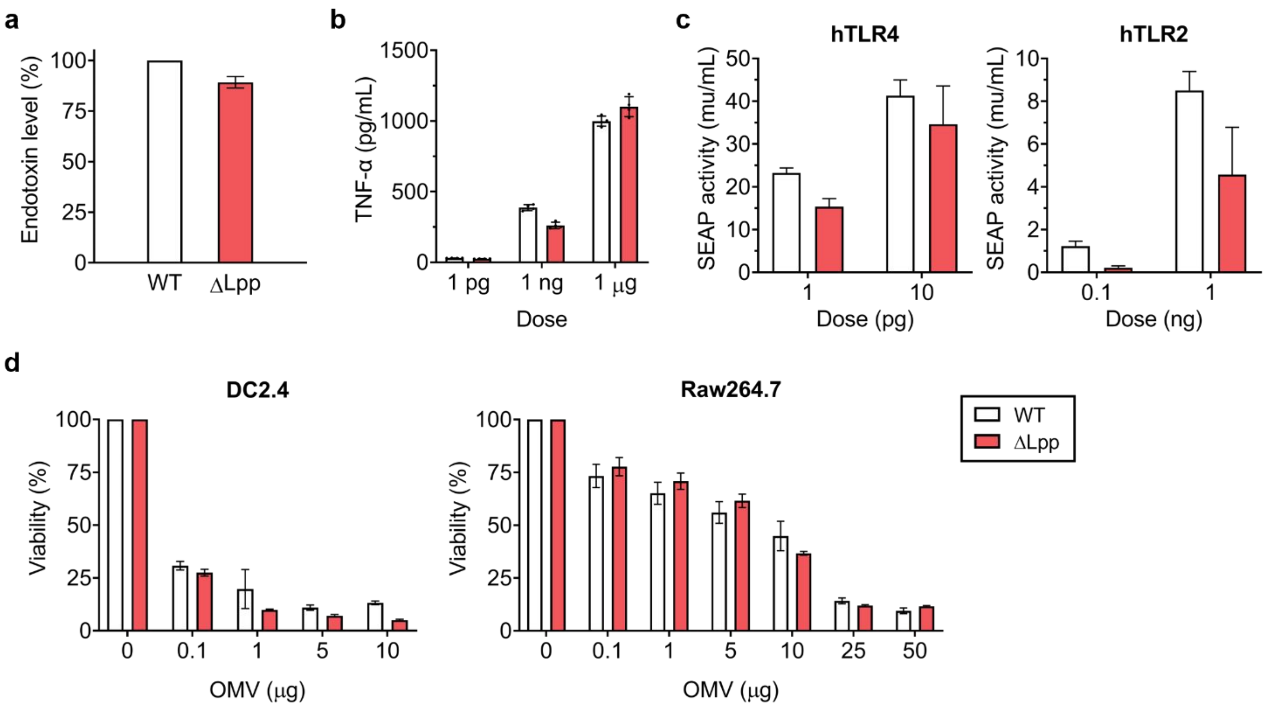

Figure S2: **The comparison of wild-type OMV (derived from BL21(DE3) strain) and ΔLpp OMV (derived from BL21(DE3) ΔLpp strain) to cytokine release, hTLR4 and hTLR2 signaling, and cytotoxicity.** (a) The endotoxin level of WT and ΔLpp OMV. (b) DC2.4 cells secreted TNF-α upon OMV stimulation after 24hrs. The secreted TNF-α were detected by ELISA kit. (c) OMV activated hTLR4 (left panel) and hTLR2 (right panel) signaling. The hTLR4 and hTLR2 activity were measured by SEAP assay. (d) The viability of DC2.4 cells and raw264.7 cells post 48hr OMV treatment. The viability was determined by CCK8 assay.

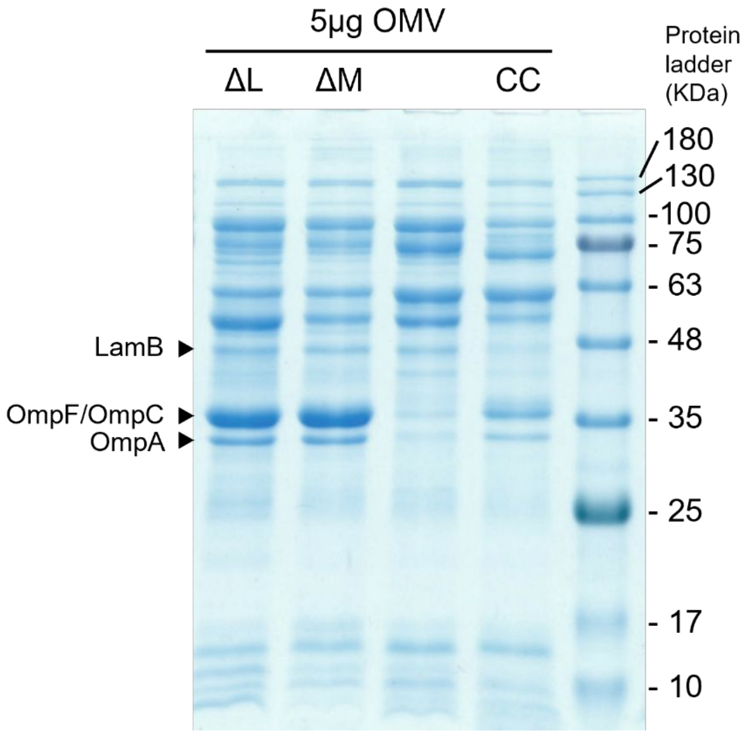

Figure S3: **SDS-PAGE of OMV variants.** The protein profile of ΔL and ΔM OMV were similar. OMV were quantified by BCA assay and loaded onto 12% SDS-PAGE. Each lane contained 5 μg OMV. Four major outer membrane proteins were indicated by arrows: LamB (47.3 kDa), OmpF (38.3 kDa), OmpC (37.1 kDa), and OmpA (35.2kDa).

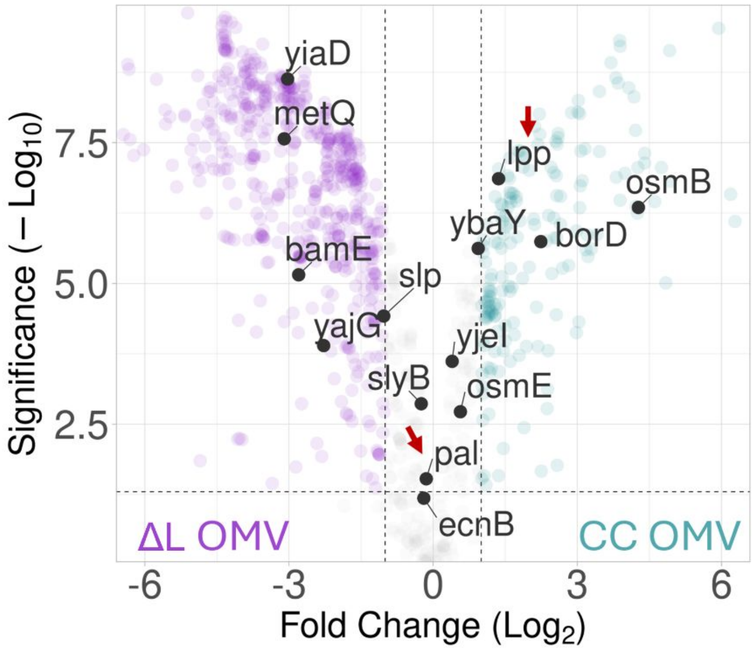

Figure S4: **Volcano plot of ΔL and CC OMV**. Volcano plot comparing the protein abundance between ΔL and CC OMV, highlighting the top 10 lipoproteins identified in ΔL OMVs. Red arrows indicate potent TLR2 agonists reported in the literature. Horizontal dashed lines represent the p-value threshold of <0.05.

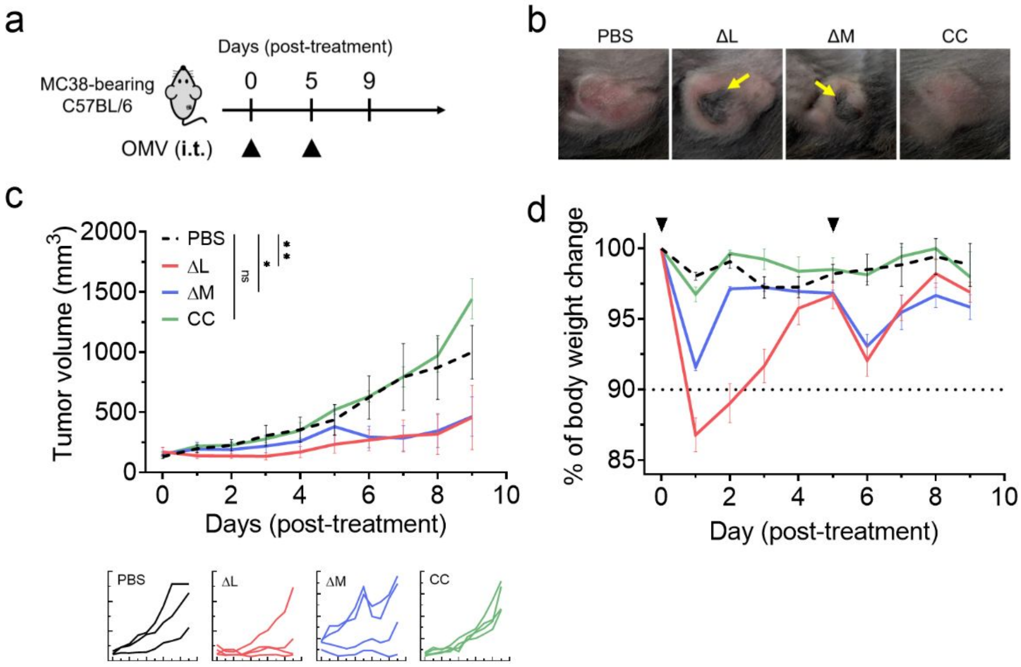

Figure S5: **The anticancer effects of LPS-free OMVs administered intratumorally are comparable to those observed with intravenous administration.** (a) Experimental design of mouse experiment to evaluate OMVs anti-tumor effects. C57BL/6 mice were subcutaneously inoculated with murine colon cancer MC38 cells. The treatment of PBS or indicated OMVs (5 μg) was delivered via intratumoral injection twice with 5-day intervals when the volume of the tumor reached about 150 mm^3. (b) The represented images of mice MC38 tumors after 1-day OMV treatment. Yellow arrow indicates scab on mice tumor. (c) The volume of mice tumor received PBS or indicated OMVs treatments was serially measured with a caliper. The data are present as the mean ± SEM. Statistical significance was calculated via two-way ANOVA with a Tukey's multiple comparisons test. ***p*<0.01, **p*<0.05; ns, not significant. The individual tumor growth curve of PBS or OMVs treated mice were showed in lower panel. (d) Mouse weight changes was serially recorded during the experiment.

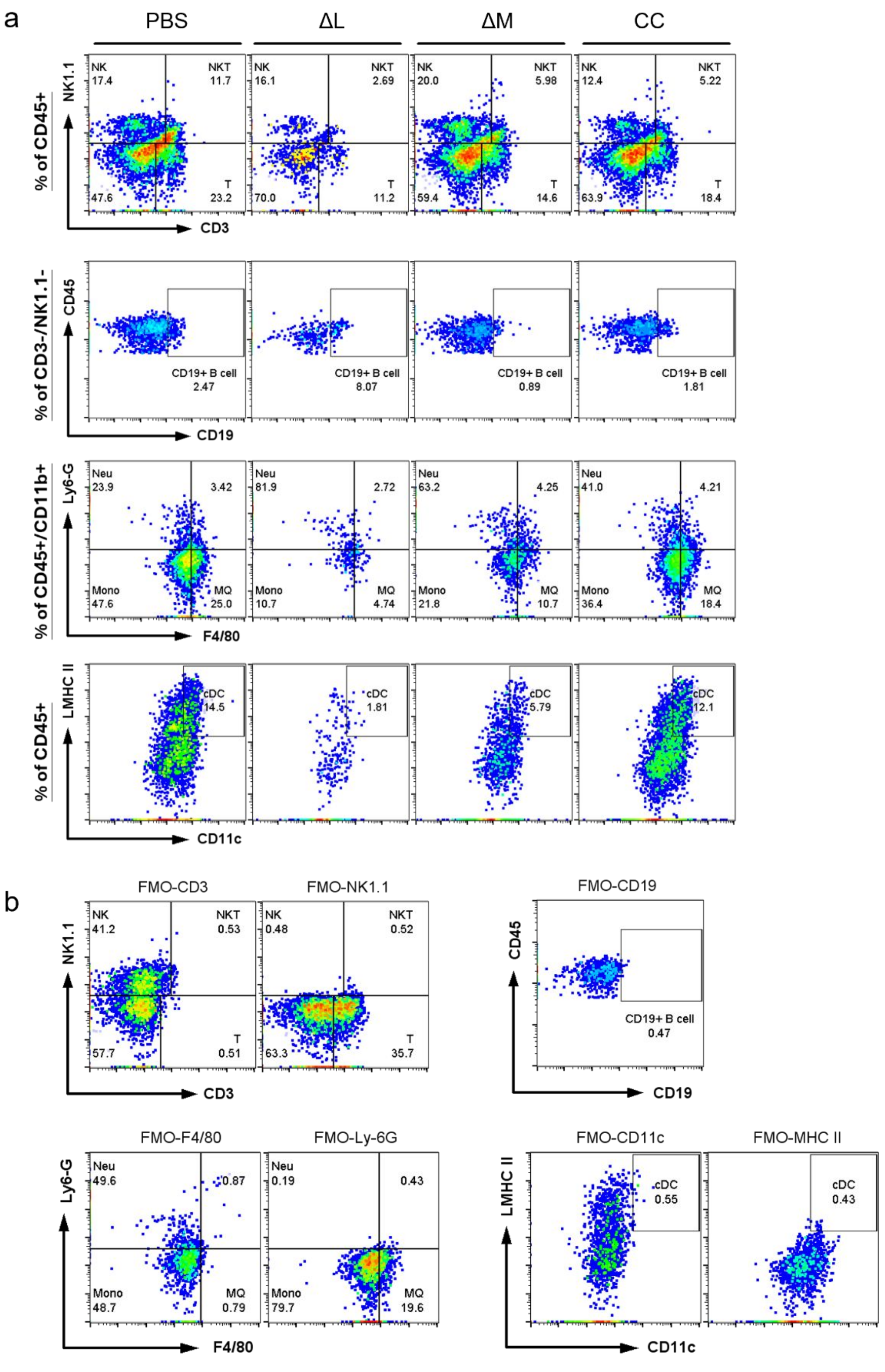

Figure S6: **Flow cytometric analysis of TIICs**. (a) PBS or indicated OMVs (5 µg) were directly injected into MC38 tumor, and the tumors were harvested after 48 hours for analysis. (b) The fluorescence minus one (FMO) for each immune cell subtype gating.

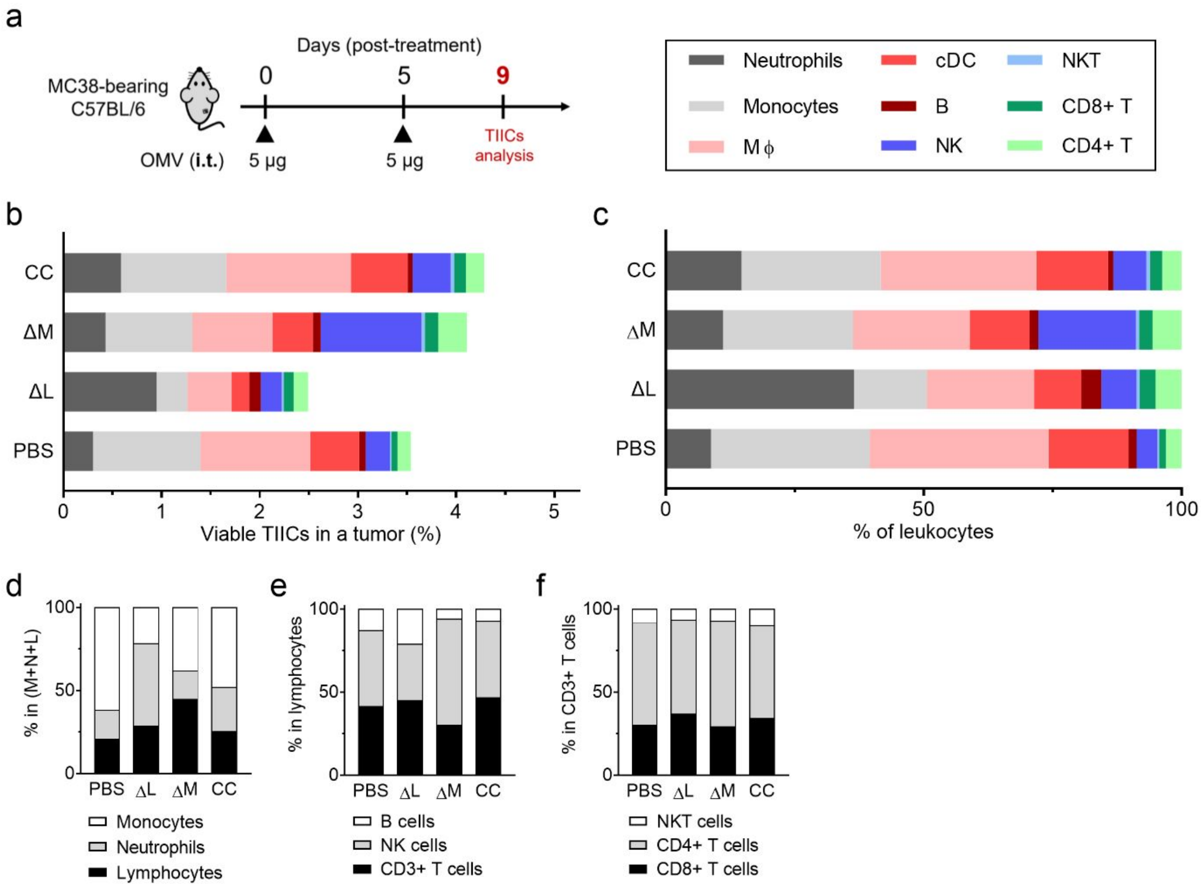

Figure S7: **Analysis of tumor-infiltrating immune cells following intratumoral treatment with OMV variants.** This analysis mirrors the procedure used in Figure 3 but was conducted at a different time point. (a) Schematic diagram illustrating an MC38 tumor-bearing mouse model for intravenous treatment by OMV variants and the time point of TIIC analysis. (b) The absolute immune cell counts in tumors. (c) The percentage of TIICs in viable leukocytes. (d-g) The percentage of cell subtypes in each group for comparing the change of immune population: the population change of monocytes, neutrophils, and lymphocytes (d), lymphocytes (B cells, NK cells, and CD3+ T cells) (e), and CD3+ T cells (CD4+ T cells, CD8+ T cells, and NKT cells) (f).

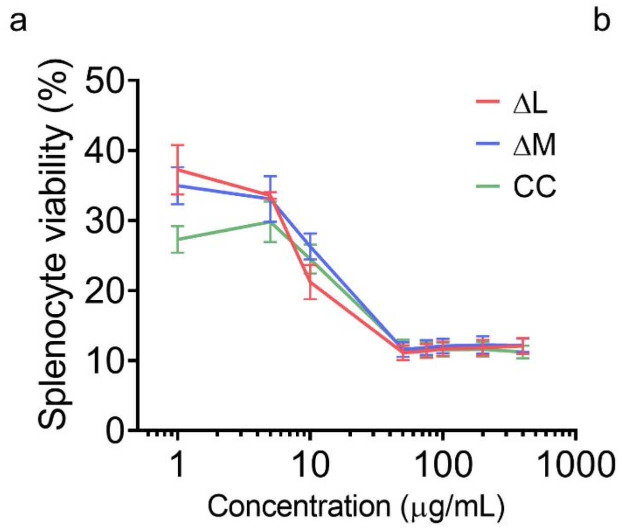

Figure S8: **The CCK8 assay for determining the viability of C57BL/6 splenocyte at different OMV variant concentration** (a) and compared to the standard, *E. coli* O111:B4 LPS (b). 100000 splenocytes were seeded in a 96-well plate, and co-incubated with OMV or LPS standard for 3 days. After the treatment, the supernatant was mixed with CCK8 agents and measured by colorimetric plate reader.

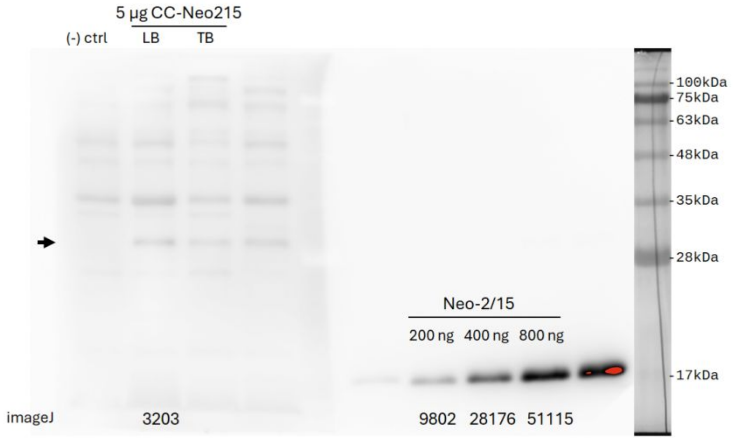

Figure S9: **Quantification of CC-Neo215 by Western blotting and ImageJ analysis.** Protein reference (Pure protein, Neo-2/15, MW=17 kDa) was loaded with 200 ng, 400 ng, 800 ng and quantified by ImageJ: 9802, 28176, 51115. The expression of Neo-2/15 on CC OMVs was evaluated.

Supplementary Tables

Supplementary Tables 1. **Top 10 proteins identified in ΔL OMVs**

No.	Protein ID	Gene	Description	Protein Probability	MaxLFQ Intensity
1	P02931	ompF	Outer membrane porin F	1	1.35E+11
2	P0A910	ompA	Outer membrane protein A	1	7.56E+10
3	P02943	lamB	Maltoporin	1	5.66E+10
4	P0A917	ompX	Outer membrane protein X	1	3.02E+10
5	P37194	slp	Outer membrane protein Slp	1	2.68E+10
6	P0ADB7	ecnB	Entericidin B	0.9998	2.56E+10
7	P0A905	slyB	Outer membrane lipoprotein SlyB	1	2.18E+10
8	P0A6F5	groEL	Chaperonin GroEL	1	1.80E+10
9	P0AEX9	malE	Maltose/maltodextrin-binding periplasmic protein	1	1.71E+10
10	P0ADA5	yajG	Uncharacterized lipoprotein YajG	1	1.19E+10

Supplementary Tables 2. **Top 10 proteins identified in CC OMVs**

No.	Protein ID	Gene	Description	Protein Probability	MaxLFQ Intensity
1	P0ABT2	dps	DNA protection during starvation protein	1	1.62E+11
2	P0A9H3	cadA	Inducible lysine decarboxylase	1	1.09E+11
3	P0A9B2	gapA	Glyceraldehyde-3-phosphate dehydrogenase A	1	7.79E+10
4	P0AFG8	aceE	Pyruvate dehydrogenase E1 component	1	7.74E+10
5	P02931	ompF	Outer membrane porin F	1	6.88E+10
6	P0A9P0	lpdA	Dihydrolipoyl dehydrogenase	1	6.85E+10
7	P0A6F5	groEL	Chaperonin GroEL	1	6.85E+10
8	P06959	aceF	Dihydrolipoyllysine-residue acetyltransferase component of pyruvate dehydrogenase complex	1	6.23E+10
9	P0A910	ompA	Outer membrane protein A	1	6.10E+10
10	P0A7Z4	rpoA	DNA-directed RNA polymerase subunit alpha	1	5.25E+10

Supplementary Tables 3. **Top 10 lipoproteins identified in ΔL OMVs**

No.	Protein ID	Gene	Description	Protein Probability	MaxLFQ Intensity
1	P37194	slp	Outer membrane protein Slp	1	2.68E+10
2	P0ADB7	ecnB	Entericidin B	0.9998	2.56E+10
3	P0A905	slyB	Outer membrane lipoprotein SlyB	1	2.18E+10
4	P0ADA5	yajG	Uncharacterized lipoprotein YajG	1	1.19E+10
5	P0ADB1	osmE	Osmotically-inducible putative lipoprotein OsmE	1	8.60E+09
6	P37665	yiaD	Probable lipoprotein YiaD	1	7.35E+09
7	P0A912	pal	Peptidoglycan-associated lipoprotein	1	6.65E+09
8	P28635	metQ	D-methionine-binding lipoprotein MetQ	1	6.51E+09
9	P0A937	bamE	Outer membrane protein assembly factor BamE	1	5.57E+09
10	P0AF70	yjeI	Uncharacterized protein YjeI	1	5.39E+09

Supplementary Tables 4. **Top 10 lipoproteins identified in CC OMVs**

No.	Protein ID	Gene	Description	Protein Probability	MaxLFQ Intensity
1	P0ADA7	osmB	Osmotically-inducible lipoprotein B	1	2.62E+10
2	P0A905	slyB	Outer membrane lipoprotein SlyB	1	1.07E+10
3	P37194	slp	Outer membrane protein Slp	1	7.64E+09
4	P69776	lpp	Major outer membrane lipoprotein Lpp	1	7.51E+09
5	P0ADB1	osmE	Osmotically-inducible putative lipoprotein OsmE	1	7.42E+09
6	P77330	borD	Prophage lipoprotein Bor homolog	1	7.15E+09
7	P0ADB7	ecnB	Entericidin B	0.9998	4.34E+09
8	P0AF70	yjeI	Uncharacterized protein YjeI	1	4.15E+09
9	P0A912	pal	Peptidoglycan-associated lipoprotein	1	3.52E+09
10	P77717	ybaY	Uncharacterized lipoprotein YbaY	1	2.85E+09

Supplementary Tables 5. **Whole blood count (CBC) analysis of 2-day OMV treatment by intravenous administration (15μg).** The CBC data indicates that the ΔL OMV group mice exhibit significant hematological abnormalities, including leukopenia, neutropenia, and thrombocytopenia. Elevated inflammation indicated by higher NLR, PLR, and SIRI values suggests higher systemic inflammation in ΔL OMV treatment.

	RBC (M/uL)	HGB (g/dL)	HCT(%)	PLT(K/uL)	PCT(%)	WBC (K/uL)	NEUT (K/uL)	LYMPH (K/uL)	MONO (K/uL)	NEUT (%)	LYMPH (%)	MONO (%)	NLR*	PLR**	SIRI***
PBS-1	8.17	12	41	897	0.7	4.1	0.95	2.86	0.22	23.1	69.8	5.4	0.33	314	0.073
PBS-2	7.98	11.5	40.1	813	0.65	3.12	0.64	2.22	0.21	20.5	71.2	6.7	0.29	366	0.061
PBS-3	8.33	12.1	42.4	849	0.67	2.72	0.4	2.14	0.11	14.7	78.7	4	0.19	397	0.021
ΔL-1	7.85	11.6	39.4	152	0.14	0.68	0.2	0.29	0.18	29.4	42.6	26.5	0.69	524	0.124
ΔL-2	6.21	9	30.9	368	0.47	0.77	0.28	0.39	0.09	36.4	50.6	11.7	0.72	944	0.065
ΔL-3	7.74	11.2	37.7	208	0.19	1.32	0.8	0.28	0.24	60.6	21.2	18.2	2.86	743	0.686
ΔM-1	7.19	10.3	35.2	240	0.22	2.55	0.52	1.72	0.29	20.3	67.5	11.4	0.3	140	0.088
ΔM-2	4.68	7.1	24.8	386	0.71	0.64	0.3	0.23	0.11	46.9	35.9	17.2	1.3	1678	0.144
ΔM-3	8.64	12.4	43.3	227	0.2	0.97	0.55	0.3	0.11	56.8	30.9	11.3	1.83	757	0.202
CC-1	8.66	12.6	42.9	417	0.35	1.61	0.51	0.91	0.18	31.7	56.5	11.2	0.56	458	0.101
CC-2	7.78	11.2	38.6	781	0.66	1.66	0.62	0.76	0.27	37.3	45.8	16.3	0.82	1028	0.220
CC-3	7.87	11.6	39.5	335	0.28	1.51	0.7	0.6	0.19	46.4	39.7	12.6	1.17	558	0.222

* NLR: Neutrophil-to-lymphocyte ratio = (Neutrophil counts / Lymphocyte counts)
** PLR: Platelet-to-lymphocyte ratio = (Platelet counts / Lymphocyte counts)
*** SIRI: Systemic inflammation response index = (Neutrophil counts×Monocyte counts) / (Lymphocyte counts)

Supplementary Tables 6. Antibodies list

Antibodies	Supplier	No. Catalog	Experiments
eBioscience™Fixable Viability Dye eFluor™780	Thermo	65-0865-14	APC, TIICs, splenocyte
anti-mouse CD16/32 antibody	Biolegend	101301	APC, TIICs, splenocyte
Pacific blue-anti-mouse CD45	Biolegend	103125	APC
Alexa Fluor 700-anti-mouse F4/80	Biolegend	123130	APC
FITC-anti-mouse I-A/I-E (MHC II)	Biolegend	107606	APC, TIICs
APC-anti-mouse CD80	BD Pharmingen	560016	APC
PE-anti-mouse CD86	BD Pharmingen	553692	APC
Alexa Fluor 700- anti-mouse CD19	Biolegend	115528	APC, TIICs
Alexa Fluor 594-anti-mouse CD11c	Biolegend	117346	APC, TIICs
PerCP/Cyanine5.5 anti-mouse CD45 antibody	Biolegend	103132	TIICs, splenocyte
Alexa Fluor 594 anti-mouse CD3 antibody	Biolegend	100240	TIICs, splenocyte
FITC anti-mouse CD4 antibody	Biolegend	100510	TIICs, splenocyte
Pacific Blue anti-mouse CD8a antibody	Biolegend	100725	TIICs, splenocyte
APC anti- mouse NK-1.1 antibody	Biolegend	108710	TIICs, splenocyte
Pacific Blue anti-mouse CD11b antibody	Biolegend	101224	TIICs, splenocyte
Alexa Fluor 647 anti-mouse Ly-6G antibody	Biolegend	127610	TIICs, splenocyte
Brilliant Violet 421 anti-mouse F4/80 antibody	Biolegend	123131	TIICs, splenocyte
